# Supplementary material for: Influence of Cold Environments on Growth, Antioxidant Status, Immunity and Expression of Related Genes in Lambs
Source: Animals (Basel). 2022 Sep 22;12(19):2535. doi: 10.3390/ani12192535 (PMC9559294; doi:10.3390/ani12192535)
Supplement: Supplementary file 1 [file animals-12-02535-s001.zip › animals-1863002-supplementary.pdf]

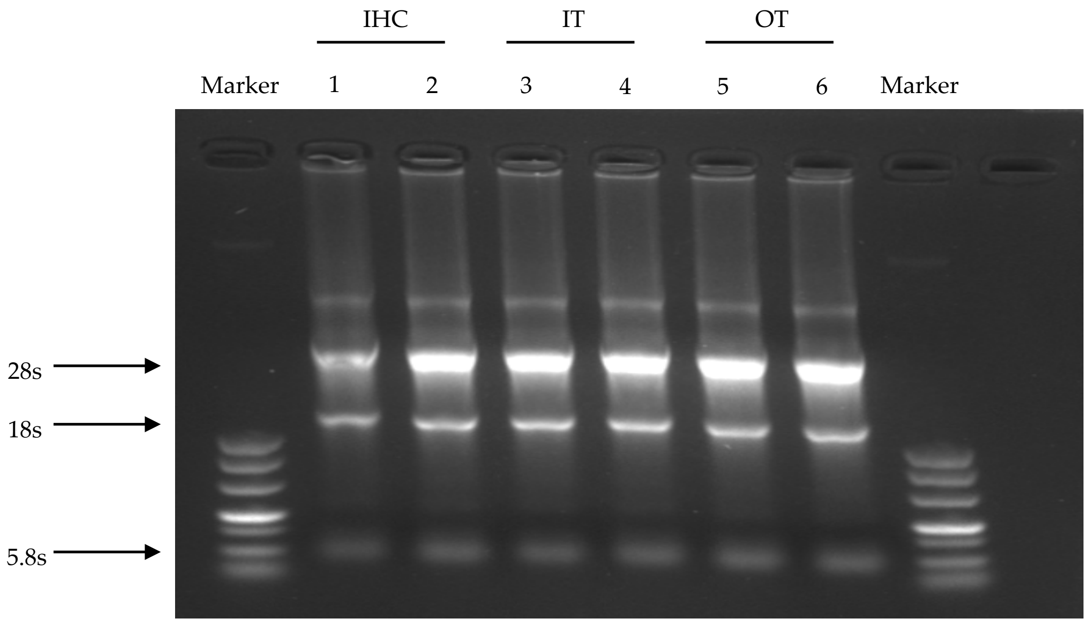

Supplementary Figure S1. Detection of total RNA integrity. Note: IHC group, indoor heating control group; IT group, indoor temperature group; OT group, outdoor temperature group.
